# Mechanism of Action of Green-Synthesized Silver Nanoparticle-Incorporated Dental Varnish Against Candida albicans

**DOI:** 10.7759/cureus.69353

**Published:** 2024-09-13

**Authors:** Joshitha Subramaniam, Remmiya Mary Varghese, Aravind Kumar Subramanian, Rajeshkumar Shanmugam

**Affiliations:** 1 Dentistry, Saveetha Dental College and Hospitals, Saveetha Institute of Medical and Technical Sciences, Saveetha University, Chennai, IND; 2 Orthodontics and Dentofacial Orthopedics, Saveetha Dental College and Hospitals, Saveetha Institute of Medical and Technical Sciences, Saveetha University, Chennai, IND; 3 Nanobiomedicine, Saveetha Dental College and Hospitals, Saveetha Institute of Medical and Technical Sciences, Saveetha University, Chennai, IND

**Keywords:** antifungal activity, candida albicans, dental varnish, green-synthesized silver nanoparticles, ocimum tenuiflorum

## Abstract

Introduction: *Candida albicans*, a common fungal pathogen, is often associated with oral infections such as denture stomatitis. Dental varnishes, especially those incorporating antimicrobial agents, have shown promise in preventing *C. albicans* colonization. This study investigates the antifungal efficacy of a dental varnish incorporating green-synthesized silver nanoparticles (AgNPs) using *Ocimum gratissimum* and *Ocimum tenuiflorum* extracts.

Materials and methods: AgNPs were synthesized via a green synthesis method using *Ocimum* extracts. The AgNPs were then incorporated into a dental varnish. The antifungal efficacy of the AgNP-incorporated varnish was evaluated against *C. albicans* using various assays, including agar well diffusion, time-kill curve, protein leakage, and cytoplasmic leakage analyses.

Results: The AgNP-incorporated dental varnish demonstrated significant antifungal activity against *C. albicans*. The agar well diffusion assay showed a dose-dependent increase in the zone of inhibition, with the highest concentration (100 µg/mL) achieving a zone of 23 mm. The time-kill curve assay indicated a concentration-dependent reduction in colony-forming units (CFU) of *C. albicans*, with the highest concentration resulting in a CFU reduction to below 10^3^ within five hours. Both protein and cytoplasmic leakage analyses confirmed membrane disruption, showing increased optical density readings at higher concentrations of AgNPs.

Discussion: The results suggest that the antifungal activity of the AgNP-incorporated dental varnish is mediated through multiple mechanisms, including membrane disruption, increased permeability leading to protein and cytoplasmic leakage, and possibly the generation of reactive oxygen species (ROS). The varnish's efficacy was comparable to that of commercial antifungal dental varnishes, highlighting its potential as a viable alternative in dental applications.

Conclusion: Green-synthesized AgNPs, when incorporated into dental varnish, exhibit potent antifungal activity against *C. albicans*. The study demonstrates that this approach can effectively disrupt fungal cells, suggesting its potential use in preventing and treating oral fungal infections. Future research should explore the in vivo efficacy, safety, and long-term stability of AgNPs in dental varnish formulations.

## Introduction

Dental varnishes have emerged as a promising method for preventing colonization by *Candida albicans*, a common fungal pathogen linked to various oral infections, including denture stomatitis [1]. Research has shown that specific varnish formulations can significantly inhibit *C. albicans* adhesion and biofilm formation, which are crucial steps in developing oral infections [2]. Previous studies have highlighted the effectiveness of certain varnishes, such as MI Varnish (GC International, Houston, Texas), in reducing *C. albicans* counts in children more effectively than other varnishes over extended periods. Additionally, the incorporation of antimicrobial agents and surface modifications in dental materials, such as vanillin in PMMA (polymethyl methacrylate) resin, has demonstrated a marked reduction in fungal adhesion [3]. These findings suggest that optimizing the surface characteristics of dental varnishes could enhance their antifungal properties, making them valuable tools in oral healthcare [4,5].

Silver nanoparticles (AgNPs) have emerged as a promising antifungal agent against *C. albicans*, particularly in dental applications. Their incorporation into dental varnishes offers an innovative approach to managing oral fungal infections. AgNPs exhibit significant antifungal activity, with studies showing minimum inhibitory concentrations (MIC) as low as 16 μg/mL for certain engineered nanomaterials. Combining AgNPs with other substances, such as calcium hydroxide, has further enhanced their efficacy, significantly reducing *C. albicans* viability. Research also indicates that increasing AgNP concentrations in denture materials correlates with reduced fungal growth, underscoring their potential use in acrylic dentures. The antifungal mechanism of AgNPs involves disrupting *C. albicans* membrane integrity and affecting biofilm formation, with transcriptomic studies revealing impacts on gene expression related to cell wall integrity and virulence.

The synergistic effects of combining *Ocimum gratissimum* and *Ocimum tenuiflorum* extracts in the synthesis of AgNPs have gained significant attention due to their potential antifungal and antimicrobial activities [6]. Using these herbal extracts for AgNP synthesis offers an environmentally friendly and cost-effective approach, with the green synthesis method involving the reduction of silver ions to nanoparticles [7]. Studies have demonstrated that these synthesized nanoparticles exhibit substantial antifungal activity against pathogens such as *C. albicans*, showing notable zones of inhibition (ZOI) [8].

The combination of extracts from both *Ocimum* species enhances the antimicrobial properties of the nanoparticles, leveraging the phytochemicals present, such as flavonoids and phenolic compounds, which improve stability and broaden their efficacy [9]. The antimicrobial action is attributed to the ability of AgNPs to disrupt microbial cell membranes, causing cell death, with the phytochemicals serving as reducing and capping agents to enhance the nanoparticle stability and reactivity. This combination of *Ocimum* extracts in AgNP synthesis presents a promising approach for enhancing antifungal efficacy, providing a foundation for future research to optimize their therapeutic potential [10].

In the present study, AgNPs were synthesized using *Ocimum gratissimum* and *Ocimum tenuiflorum* extracts, and its based dental varnish was formulated. The antimicrobial properties of the AgNP-incorporated dental varnish were evaluated using various assays, including the agar well diffusion technique, time-kill curve assay, protein leakage assay, and cytoplasmic leakage analysis.

## Materials and methods

Preparation of *Ocimum tenuiflorum* and *Ocimum gratissimum* herbal formulation

Fresh leaves of *Ocimum tenuiflorum* and *Ocimum gratissimum* were collected and thoroughly washed with distilled water to eliminate any surface contaminants. The leaves were then shade-dried at ambient room temperature until completely dehydrated. Once dried, the leaves were finely ground into a powder using a mechanical grinder. To prepare the extract, 1 g of each powdered leaf was combined with 100 mL of distilled water. The resulting mixture was subjected to heating at 60°C for 15-20 minutes using a heating mantle. Following the heating process, the mixture was filtered gradually using filter paper to obtain a clear extract.

Synthesis of *Ocimum tenuiflorum* and *Ocimum gratissimum* herbal formulation-mediated AgNPs and its base dental varnish

For the green synthesis of AgNPs, a 1 mM silver nitrate solution was prepared by dissolving silver nitrate in 80 mL of distilled water. Subsequently, 20 mL of the filtered herbal extract formulation was added to the silver nitrate solution. The resulting mixture was centrifuged at 8000 rpm for 10 minutes to facilitate nanoparticle formation and separation. The pellet obtained post-centrifugation was collected and stored for subsequent use in dental varnish preparation. To formulate the dental varnish incorporating *Ocimum tenuiflorum* and *Ocimum gratissimum-*based AgNPs, 7 mL of a chitosan solution was combined with 2.5 mL of ethanol and 500 µL of the synthesized AgNPs. The mixture was thoroughly blended using a vortex mixer for 1-2 hours to ensure a homogeneous distribution of AgNPs throughout the varnish matrix.

Agar well diffusion technique

The antifungal activity of the AgNP-incorporated dental varnish was evaluated using the agar well diffusion method. Mueller-Hinton agar plates were prepared and inoculated with a standardized inoculum of *C. albicans* (approximately 106 colony-forming units (CFU)/mL). Wells of 9 mm diameter were punched into the agar using a sterile cork borer, and the dental varnish containing different concentrations of AgNPs was added into the respective wells. A commercial dental varnish was used as a standard. The plates were incubated at 37°C for 24 hours, and the ZOI around the wells was measured in millimeters.

Time-kill curve assay

The time-kill curve assay was conducted to evaluate the rate of fungal cell reduction over time. A standardized inoculum of *C. albicans* (approximately 106 CFU/mL) was treated with AgNP-incorporated dental varnish at concentrations of 25 µg/mL, 50 µg/mL, and 100 µg/mL, along with a commercial dental varnish (standard) and an untreated control. Aliquots were collected at 0, 1, 2, 3, 4, and 5 hours, serially diluted, and their optical density (OD) was measured at 600 nm using an enzyme-linked immunosorbent assay (ELISA) plate reader to track changes in microbial load over time.

Protein leakage analysis

To assess cell membrane integrity, protein leakage analysis was performed by measuring the OD at 280 nm of the supernatant from treated fungal suspensions. *C. albicans* cells were exposed to AgNP-incorporated dental varnish at varying concentrations (25 µg/mL, 50 µg/mL, and 100 µg/mL), a commercial dental varnish, and a control (without AgNPs) for five hours. Samples were centrifuged at 5000 rpm for 10 minutes, and the OD of the supernatant was measured using a UV-visible spectrophotometer. An increase in OD indicated protein leakage due to cell membrane disruption.

Cytoplasmic leakage analysis

Cytoplasmic leakage was evaluated by measuring the OD at 260 nm, corresponding to nucleic acids and other cytoplasmic contents. *C. albicans* cells were treated with different concentrations of AgNP-incorporated dental varnish, a commercial varnish, and a control (without AgNPs). After incubation for five hours, samples were centrifuged, and the OD of the supernatant was measured at 260 nm using a UV-visible spectrophotometer. Higher OD values indicated increased cytoplasmic leakage, suggesting membrane damage.

## Results

Antifungal activity

**Figure 1:**
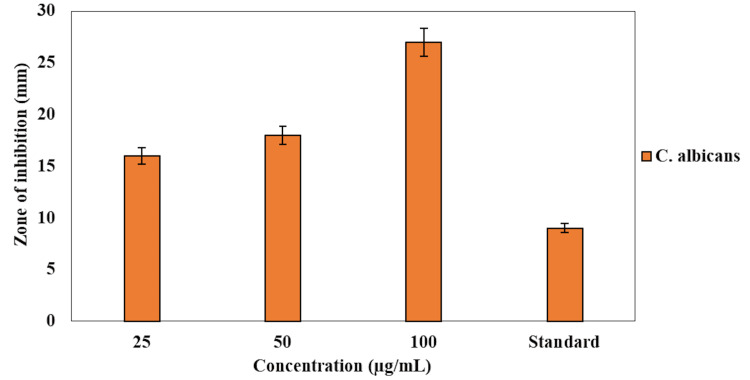
Antimicrobial activity of the dental varnish incorporated with AgNPs against Candida albicans. The graph shows the zone of inhibition (mM) at different concentrations of the dental varnish and the standard. AgNPs: silver nanoparticles

The antifungal activity of the dental varnish incorporated with green-synthesized AgNPs against *C. albicans* was evaluated using the agar well diffusion method. The ZOI was measured at different concentrations of the dental varnish, as shown in Figure 1. The results demonstrated a dose-dependent increase in antifungal activity. At the lowest concentration of 25 µg/mL, the dental varnish exhibited a ZOI of 15 mm. Increasing the concentration to 50 µg/mL resulted in a ZOI of 19 mm, while the highest concentration of 100 µg/mL achieved a ZOI of 23 mm, indicating significant antifungal activity against *C. albicans*. The commercial dental varnish, used as a standard, exhibited a comparatively smaller ZOI of 10 mm. These results suggest that the incorporation of AgNPs into the dental varnish enhances its antifungal efficacy against *C. albicans*, with the effectiveness increasing with the concentration of AgNPs.

Time-kill curve assay

**Figure 2:**
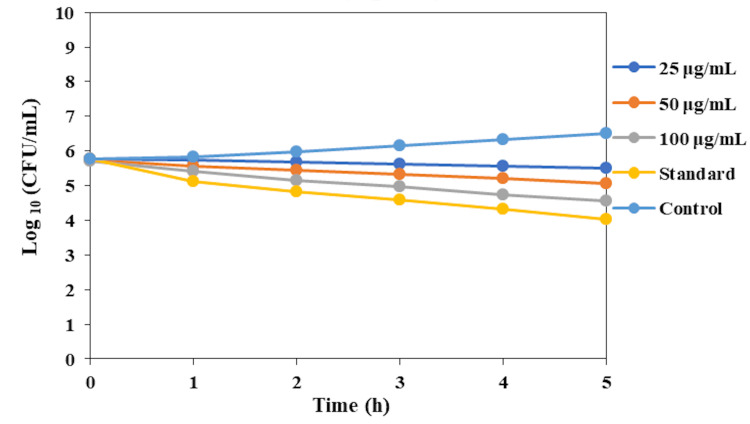
Time-kill curve of the dental varnish incorporated with green-synthesized silver nanoparticles (AgNPs) against Candida albicans. The graph shows the log reduction in CFU/mL over time at different concentrations of AgNPs and the standard control. CFU: colony-forming units

The time-kill curve assay was conducted to evaluate the fungicidal effect of dental varnish incorporated with green-synthesized AgNPs against *C. albicans* over a period of five hours. The assay measured the reduction in CFU per mL at various time intervals, as depicted in Figure 2.

The results indicated that the AgNP-incorporated dental varnish exhibited a concentration-dependent fungicidal activity. At the initial time point (0 hours), the *C. albicans* CFU/mL was approximately 106 for all samples. At a 25 µg/mL concentration, the CFU/mL slightly decreased over time, reaching approximately 105 after five hours. The varnish containing 50 µg/mL of AgNPs demonstrated a more pronounced reduction in CFU/mL, with a consistent decrease over the five-hour period, ending at approximately 104 CFU/mL. The highest concentration of 100 µg/mL showed the most significant fungicidal effect, reducing the CFU/mL to below 103 within five hours. The commercial dental varnish, used as a standard, exhibited a similar trend, achieving a reduction to approximately 103 CFU/mL by the five-hour mark.

The control (without AgNPs) maintained a relatively stable CFU/mL, indicating minimal fungicidal activity. These results demonstrate that the dental varnish containing green-synthesized AgNPs effectively inhibits the growth of *C. albicans* in a time- and concentration-dependent manner, with the highest concentration (100 µg/mL) being the most effective. The fungicidal activity of the varnish is comparable to that of the commercial dental varnish, suggesting its potential as a viable alternative in dental applications.

Protein leakage analysis

**Figure 3:**
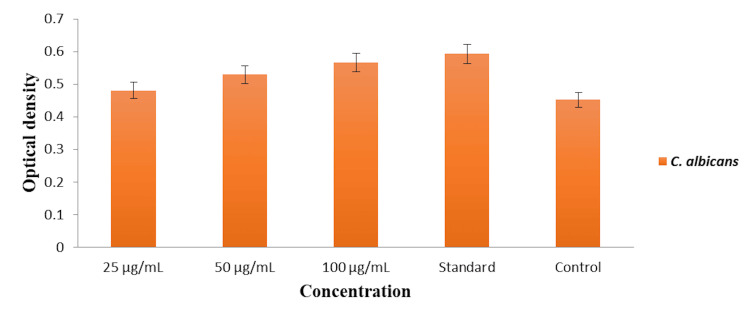
Protein leakage analysis of the dental varnish incorporated with green-synthesized silver nanoparticles (AgNPs) against Candida albicans. The graph shows the optical density (OD) at 280 nm, indicating the amount of protein leakage from fungal cells at different concentrations of AgNPs and the standard control.

The effect of the dental varnish incorporated with green-synthesized AgNPs on protein leakage from *C. albicans* cells was evaluated by measuring the OD of the extracellular protein content. The results, as depicted in Figure 3, show the OD at 280 nm, corresponding to the concentration of proteins leaked from the fungal cells at various concentrations of the AgNPs. At the lowest concentration of 25 µg/mL, the OD was recorded at approximately 0.45, indicating a moderate level of protein leakage. Increasing the concentration to 50 µg/mL resulted in a slight increase in OD to approximately 0.5, suggesting enhanced membrane damage and subsequent protein leakage. The highest concentration of 100 µg/mL showed the most significant protein leakage, with an OD of approximately 0.55. The commercial dental varnish displayed a similar level of protein leakage, with an OD of approximately 0.55, comparable to the 100 µg/mL concentration of AgNPs. The control (without AgNPs) exhibited the lowest OD, approximately 0.4, indicating minimal protein leakage and intact cell membranes.

These findings suggest that the dental varnish incorporated with AgNPs induces membrane damage in *C. albicans*, leading to increased protein leakage. The extent of this leakage is concentration-dependent, with higher concentrations of AgNPs causing more significant disruption of the fungal cell membrane. This disruption is comparable to that induced by the standard antifungal treatment, highlighting the potential of AgNPs as an effective antifungal agent in dental applications.

Cytoplasmic leakage analysis

**Figure 4:**
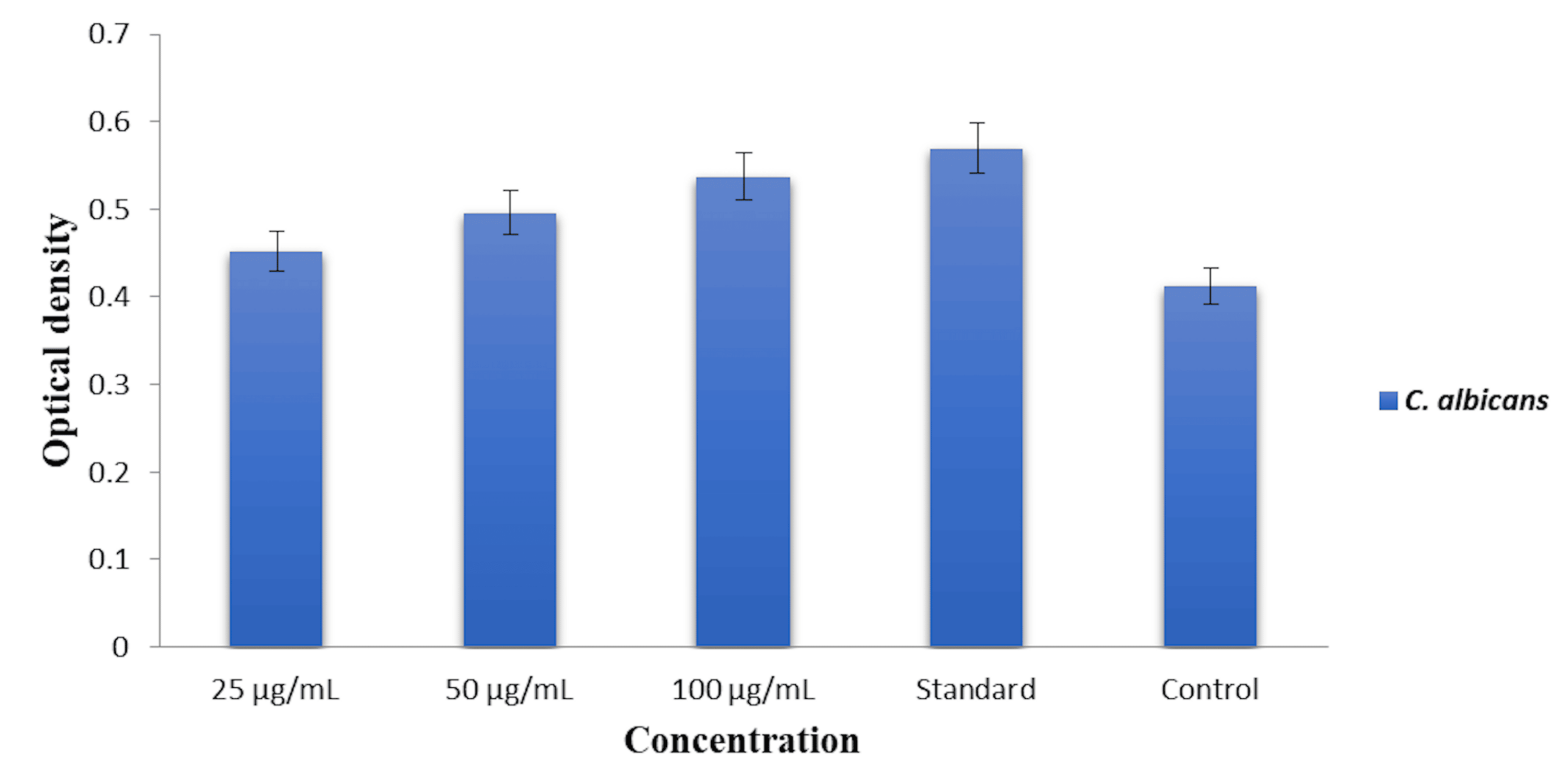
Cytoplasmic leakage analysis of the dental varnish incorporated with green-synthesized silver nanoparticles (AgNPs) against Candida albicans. The graph shows the optical density (OD) at 260 nm, indicating the amount of cytoplasmic leakage at different concentrations of AgNPs and the standard control.

The effect of dental varnish incorporated with green-synthesized AgNPs on cytoplasmic leakage from *C. albicans* cells was assessed by measuring the OD at 260 nm, which corresponds to the amount of nucleic acids and other cytoplasmic contents released from the cells. The results are presented in Figure 4.

At a 25 µg/mL concentration, the OD was measured at approximately 0.45, indicating a moderate level of cytoplasmic leakage. The OD slightly increased to 0.48 when the concentration of AgNPs was raised to 50 µg/mL, showing enhanced membrane disruption and subsequent cytoplasmic leakage. The highest concentration of 100 µg/mL resulted in the most significant cytoplasmic leakage, with an OD of approximately 0.52. The commercial dental varnish used as a control displayed an OD of approximately 0.54, which is comparable to the leakage observed at the 100 µg/mL concentration of AgNPs.

The control (without AgNPs) showed the lowest OD of approximately 0.4, indicating minimal cytoplasmic leakage and suggesting intact cellular integrity in the absence of the nanoparticles. These findings indicate that the dental varnish incorporated with AgNPs induces a concentration-dependent increase in cytoplasmic leakage from *C. albicans* cells. The highest level of leakage was observed at the 100 µg/mL concentration, which was comparable to the effect of the commercial dental varnish. This indicates that the AgNPs are capable of disrupting the fungal cell membrane, leading to the release of cytoplasmic contents, which is indicative of cell damage.

## Discussion

The current study explored the antifungal efficacy and underlying mechanism of action of a dental varnish incorporated with green-synthesized AgNPs against *C. albicans*, a pathogenic yeast commonly associated with oral infections such as oral candidiasis. The findings from this study highlight the potential of AgNPs as an effective antifungal agent in dental applications. The results from various assays, including the time-kill curve, protein leakage, and cytoplasmic leakage analyses, provide a comprehensive understanding of how AgNPs exert their antifungal effects on *C. albicans*. The observed antifungal activity can be attributed to multiple mechanisms.

The time-kill curve assay demonstrated a significant reduction in the CFU of *C. albicans* over time, particularly at higher concentrations of AgNPs. This suggests that AgNPs disrupt the cell membrane, leading to cell death [11,12]. The increase in protein and cytoplasmic leakage observed in this study further supports this mechanism. The nanoparticles likely interact with the fungal cell membrane, causing increased permeability and ultimately leading to leakage of intracellular components, including proteins and nucleic acids [13,14].

Although not directly measured in this study, it is well-documented in the literature that AgNPs can generate reactive oxygen species (ROS) upon interaction with microbial cells. These ROS can induce oxidative stress, damaging cellular components such as lipids, proteins, and DNA and leading to cell death [15]. The enhanced antifungal activity observed at higher concentrations of AgNPs in the current study may be partially attributed to the increased generation of ROS [16].

Silver ions released from AgNPs are known to have a high affinity for sulfur-containing proteins, which are abundant in the cell membrane and cytoplasm [17]. The interaction of Ag^+^ ions with thiol groups in these proteins can lead to their inactivation, disrupting vital cellular processes such as respiration and leading to cell death. This mechanism could explain the dose-dependent increase in protein leakage observed in the study [18].

The study also compared the antifungal efficacy of the AgNP-incorporated dental varnish with a commercial dental varnish. The results showed that the AgNP varnish was as effective as, if not more effective, the standard antifungal treatment, particularly at higher concentrations. This finding suggests that AgNPs could serve as a viable alternative to conventional antifungal agents, which is particularly important in the context of rising antifungal resistance [19].

Implications for dental applications

The incorporation of green-synthesized AgNPs into dental varnish offers a promising approach to managing oral infections caused by *C. albicans*. The eco-friendly synthesis of these nanoparticles using plant extracts not only provides a sustainable alternative to conventional chemical synthesis methods but also enhances the biocompatibility of the resulting nanoparticles [20]. This is crucial for dental applications, where the safety and efficacy of materials used in the oral cavity are of paramount importance. Furthermore, the broad-spectrum antimicrobial properties of AgNPs, coupled with their ability to be easily incorporated into various dental materials, make them an attractive option for developing multifunctional dental products. The use of AgNPs in dental varnish could potentially prevent or treat oral candidiasis while also offering additional protection against other oral pathogens [21].

Limitations and future directions

While the study provides valuable insights into the antifungal mechanism of AgNPs, several limitations need to be addressed in future research. First, the exact biochemical pathways through which AgNPs exert their effects on *C. albicans* remain to be fully elucidated. Advanced molecular techniques, such as transcriptomic and proteomic analyses, could provide a deeper understanding of these mechanisms. Second, the study was conducted in vitro, and the in vivo efficacy and safety of the AgNP-incorporated dental varnish need to be evaluated. Animal studies and clinical trials are necessary to confirm the potential of AgNPs for use in dental practice. Finally, the long-term stability and potential cytotoxicity of AgNPs in the oral environment should be thoroughly investigated. While the current study indicates that AgNPs are effective against *C. albicans*, their impact on oral tissues, beneficial oral microbiota, and overall oral health must be considered.

## Conclusions

The green-synthesized AgNPs incorporated into dental varnish exhibit potent antifungal activity against *C. albicans*, primarily through mechanisms involving membrane disruption, protein interaction, and potentially ROS generation. The findings suggest that AgNPs could serve as an effective alternative to traditional antifungal agents in dental applications, offering a sustainable and biocompatible option for preventing and treating oral candidiasis. However, further research is needed to fully understand the mechanisms at play and to ensure the safety and efficacy of AgNPs in clinical settings.

## References

[1] Your AM (2024). Experimental substantiation of the effectiveness of antimicrobial varnish for the treatment of finished prostheses in the prevention of prosthetic stomatitis. Rep of Vinnytsia Nation Med Univ.

[2] Ramya Jr, Gupta DN, Gambhir DN (2022). Comparative evaluation of effect of different varnishes (MI varnish, Clinpro varnish and Fluor protector varnish) On Candida albicans count and salivary PH in children aged 6-12 years - a randomized control trial. J Pharm Negat.

[3] Thaweboon S, Thaweboon B, Sopavanit C (2023). Candidal adhesion to an oral obturator PMMA resin incorporated with vanillin. Key Eng Mater.

[4] El Zawawy NA, El-Safty S, Kenawy ER, Ibrahim Salem S, Ali SS, Mahmoud YA (2023). Exploring the biomedical potential of a novel modified glass ionomer cement against the pandrug-resistant oral pathogen Candida albicans SYN-01. J Oral Microbiol.

[5] Le PH, Linklater DP, Medina AA, MacLaughlin S, Crawford RJ, Ivanova EP (2024). Impact of multiscale surface topography characteristics on Candida albicans biofilm formation: from cell repellence to fungicidal activity. Acta Biomater.

[6] Varghese RM, S AK, Shanmugam R (2024). Antimicrobial activity of silver nanoparticles synthesized using Ocimum tenuiflorum and Ocimum gratissimum herbal formulations. Cureus.

[7] Tailor G, Yadav BL, Chaudhary J, Joshi M, Suvalka C (2020). Green synthesis of silver nanoparticles using Ocimum canum and their anti-bacterial activity. Biochem Biophys Rep.

[8] Pradeep M, Kruszka D, Kachlicki P (2022). Uncovering the phytochemical basis and the mechanism of plant extract-mediated eco-friendly synthesis of silver nanoparticles using ultra-performance liquid chromatography coupled with a photodiode array and high-resolution mass spectrometry. ACS Sustain Chem Eng.

[9] Kavitha A, Shanmugan S, Awuchi CG (2021). Synthesis and enhanced antibacterial using plant extracts with silver nanoparticles: therapeutic application. Inorg Chem Commun.

[10] Varghese RM, S AK, Shanmugam R (2024). Antimicrobial activity of zinc oxide nanoparticles synthesized using Ocimum tenuiflorum and Ocimum gratissimum herbal formulation against oral pathogens. Cureus.

[11] Abdallah BM, Ali EM (2022). Therapeutic effect of green synthesized silver nanoparticles using Erodium glaucophyllum extract against oral candidiasis: in vitro and in vivo study. Molecules.

[12] Attallah NG, Elekhnawy E, Negm WA, Hussein IA, Mokhtar FA, Al-Fakhrany OM (2022). In vivo and in vitro antimicrobial activity of biogenic silver nanoparticles against staphylococcus aureus clinical isolates. Pharmaceuticals (Basel).

[13] Alherz FA, Negm WA, Elekhnawy E, El-Masry TA, Haggag EM, Alqahtani MJ, Hussein IA (2022). Silver nanoparticles prepared using Encephalartos laurentianus de wild leaf extract have inhibitory activity against Candida albicans clinical isolates. J Fungi (Basel).

[14] Spoladori LF, Andriani GM, Castro IM (2023). Synergistic antifungal interaction between Pseudomonas aeruginosa LV strain metabolites and biogenic silver nanoparticles against Candida auris. Antibiotics (Basel).

[15] Jomova K, Raptova R, Alomar SY, Alwasel SH, Nepovimova E, Kuca K, Valko M (2023). Reactive oxygen species, toxicity, oxidative stress, and antioxidants: chronic diseases and aging. Arch Toxicol.

[16] Sies H, Belousov VV, Chandel NS (2022). Defining roles of specific reactive oxygen species (ROS) in cell biology and physiology. Nat Rev Mol Cell Biol.

[17] Khina AG, Krutyakov YA (2021). Similarities and differences in the mechanism of antibacterial action of silver ions and nanoparticles. Appl Biochem Microbiol.

[18] Ribeiro LG, Roque GS, Conrado R, De Souza AO (2023). Antifungal activity of mycogenic silver nanoparticles on clinical yeasts and phytopathogens. Antibiotics (Basel).

[19] Almeida NL, Peralta LC, Pontes FM, Rinaldo D, Porto VC, Lara VS (2024). Anti-Candida activity and biocompatibility of silver nanoparticles associated with denture glaze: a new approach to the management of denture stomatitis. Folia Microbiol (Praha).

[20] Varghese RM, S AK, Shanmugam R (2024). Cytotoxicity and characterization of zinc oxide and silver nanoparticles synthesized using Ocimum tenuiflorum and Ocimum gratissimum herbal formulation. Cureus.

[21] Kumar S, Khan HM, Khan MA (2023). Broad-spectrum antibacterial and antibiofilm activity of biogenic silver nanoparticles synthesized from leaf extract of Phyllanthus niruri. J King Saud Univ Sci.

